# Long-Term Outcome of Corneal and Anterior Chamber Angle Parameters after Combined Laser Iridotomy and Iridoplasty Using Dual Scheimpflug Analyzer: 1 Year Results

**DOI:** 10.3390/jcm11030813

**Published:** 2022-02-03

**Authors:** Hyun-kyung Cho, Wooseok Choae

**Affiliations:** 1 Department of Ophthalmology, School of Medicine, Gyeongsang National University Changwon Hospital, Gyeongsang National University, Changwon 51472, Korea; 2 lnstitute of Health Sciences, School of Medicine, Gyeongsang National University, Jinju 52727, Korea; 3 Department of Ophthalmology, Barunsungmo Eye Clinic, Busan 49247, Korea; choiooseok@hanmail.net

**Keywords:** anterior chamber angle, laser iridoplasty, laser iridotomy, primary angle-closure, dual Scheimpflug analyzer

## Abstract

Background: To investigate the outcomes of corneal and anterior chamber angle (ACA) parameters after laser iridotomy (LI) combined with peripheral iridoplasty (PI) using dual Scheimpflug analyzer in the long term. Methods: Fifty-eight eyes (58 subjects) with shallow AC were included in this prospective cohort study. Images of the Dual Scheimpflug analyzer were obtained before, 1 week, and 1 year after LI and PI. Pachymetry from three zones (central, middle, and peripheral), corneal aberration, and spherical equivalent (SE) were acquired. AC depth (ACD), AC volume (ACV), ACA from four quadrants, and intraocular pressure (IOP) were also obtained. For comparison of the results, the linear mixed-effects model was employed. Results: ACD significantly increased from 2.09 ± 0.25 mm to 2.10 ± 0.23 mm at 1 year after laser (all *p <* 0.05). ACV and ACA increased significantly after laser at 1 year (all *p <* 0.05). IOP significantly decreased from 15.97 ± 4.20 mmHg to 13.73 ± 2.63 mmHg at 1 year (all *p <* 0.0001). No significant changes were found in the coma, trefoil, total corneal aberration, pachymetry from three zones, corneal volume, central corneal thickness, and SE after LI and PI until 1 year (all *p* > 0.05). Conclusions: LI plus PI ameliorated parameters of ACA efficiently and significantly reduced IOP in eyes with shallow AC until 1 year of long-term follow-up. However, parameters of the cornea and SE were not influenced by LI with PI until after 1 year.

## 1. Introduction

Primary angle-closure glaucoma (PACG) is more prevalent in Asians than other races with regard to population-based studies [1,2]. Asians are considered to have an anatomic and genetic susceptibility to developing primary angle-closure (PAC) [3,4]. Although pupillary block is considered to be the major mechanism of PAC, other mechanisms other than a pupillary block might also contribute to PAC [5,6,7]. Laser iridotomy (LI) relieves the pupillary block by generating a shunt that allows aqueous humor flow from the posterior chamber to the anterior chamber [8,9]. LI is considered as the primary treatment means for PAC [8,9], but recently, clear-lens extraction is also considered as one of the options for first-line treatment [10]. In previous studies, the anterior chamber angle (ACA) of some PAC eyes that underwent LI remained closed, including those in our study [11,12,13,14]. Moreover, some eyes that received LI showed the deterioration of peripheral anterior synechiae (PAS) [15,16,17]. In our previous study, the quantitative changes in the peripheral ACA after LI alone and LI combined with peripheral iridoplasty (PI) were compared using the iridotrabecular contact (ITC) index [18]. The ITC index is a summary value of the total amount of angle-closure demonstrated as a percentage (i.e., extent of angle-closure/360°) [19]. We revealed that LI plus PI opened the peripheral ACA better than LI alone using the ITC index. Iridoplasty may be capable of relieving the PAC caused by mechanisms other than pupillary block. 

On the basis of our previous study results, we prefer to conduct PI when LI is performed on PAC spectrum eyes. PI produces the contraction of the iris at the periphery and pulls the iris off the ACA and, consequently, opens the peripheral ACA [20]. PI has been reported to be a relatively safe means of PAC [21,22]. Even though we employ PI carefully with the minimum laser power needed to make the contracture visible, the impact of this circumferential laser to the cornea, particularly at the periphery, has not been well assessed. 

Changes in corneal topography after management of glaucoma with surgical trabeculectomy and medical eye drops have been reported previously [23,24,25]. Corneal aberrations, particularly high-order aberrations, could influence the quality of vision [26]. In our previous study, we found no changes in the corneal topographic parameters. including corneal parameters from three zones (central, middle, and peripheral) and coma, trefoil, and total corneal wavefront aberration and at one week after LI plus PI using dual Scheimpflug analyzer. In contrast, the ACA parameters including anterior chamber depth (ACD) and intraocular pressure (IOP) improved significantly one week after LI plus PI. However, these results of our previous study were short-term observations of one week. To our knowledge, the long-term evaluation of corneal topographic parameters after LI combined with PI using a dual Scheimpflug analyzer has not been reported. Moreover, long-term changes in ACA parameters after LI in combination with PI also have not been assessed before, especially with a dual Scheimpflug analyzer. 

The Galilei topographer (Galilei G4; Ziemer Ophthalmic Systems, Port, Switzerland) applies a dual Scheimpflug camera system together with a Placido disk. The Galilei dual Scheimpflug analyzer enhances the visualization of the circumferential peripheral ACA and offers ACA parameters including mean ACA values from the entire angle. The Galilei device aligns the limbus and pupil for the correct comparison of images from previous ones [27].

In this prospective observational study, we investigated the long-term changes in corneal parameters including corneal aberrations along with the pachymetry from three zones after LI with PI using the dual Scheimpflug Analyzer up to one year. We also inspected the changes in ACA parameters and IOP after LI with PI in up to one year of follow-up.

## 2. Materials and Methods

This was a prospective observational study performed according to the tenets of the Declaration of Helsinki. The present study was approved by the Institutional Review Board of the Gyeongsang National University Changwon Hospital. Written informed consent was acquired from all participants.

### 2.1. Subjects

Patients diagnosed with primary angle closure (PAC), primary angle-closure suspect (PACS), and primary angle-closure glaucoma (PACG) who received LI plus PI at glaucoma clinic of Gyeongsang National University Changwon Hospital were enrolled in this study. PAC was determined when an eye showed a shallow ACA (appositional contact between the trabecular meshwork (TM) and peripheral iris of >270 degrees on gonioscopy) with characteristics suggesting blockage of TM by the peripheral iris such as IOP elevation, excessive pigment deposition on the TM, iris whorls (deformation of iris radial fibers), ‘‘glaukomflecken’’ lens opacity, or peripheral anterior synechiae (PAS) but absence of glaucomatous optic neuropathy or visual field (VF) defects [28]. PACS was determined as an eye with a narrow ACA (pigmented TM invisible of >270 degrees on gonioscopy), an IOP of ≤21 mmHg, and no visible PAS or glaucomatous optic disc injury [28]. PACG was defined as eyes with a narrow ACA showing glaucomatous optic disc injury (neuroretinal rim thinning with a vertical cup-to-disc ratio of 0.7 or an asymmetry between eyes of 0.2 or notching attributed to glaucoma) accompanied by corresponding VF defects [28]. Evaluation and diagnosis of PAC spectrum eyes were performed by a single glaucoma specialist (H.-k.C.).

The present study aimed to investigate the changes in ACA after LI combined with PI. Therefore, in addition to the previously described criteria, eyes with PAS before the laser procedures were also excluded in the present study [28]. Only those of newly diagnosed eyes were included. Chronic PACG with elevated IOP and showing definite PAS were excluded because those eyes may not respond to LI or PI. Furthermore, it may influence the results of the changes in the peripheral ACA or corneal parameters after LI combined with PI. Those using or having a history of topical or systemic medications that could affect pupil reflex or ACA were excluded from our study. Those with a history of ocular surgery including laser trabeculoplasty, LI, PI, or a history of cataract surgery or phacoemulsification during the follow-up period were excluded. Patients who were not able to fixate for the dual Scheimpflug analyzer were also excluded. Secondary angle-closure eyes derived from diseases, for example, uveitic glaucoma or neovascular glaucoma were excluded. After the acquisition of dual Scheimpflug analyzer images, bad images owing blinking or poor fixation were also excluded. If both eyes met the same diagnosis, only one eye was selected randomly for inclusion using the Excel random function. 

### 2.2. Laser Peripheral Iridotomy and Peripheral Iridoplasty

LI combined with PI was conducted on the same day by a single glaucoma specialist (H.-k.C.). LI was performed on the superior region of the iris (10~2 o’clock) with argon laser and subsequently by neodymium-yttrium-aluminum-garnet lasers. The eyes were applied with 2% pilocarpine 30 min before LI. An argon laser applied power of 600 to 1000 mW and 50~100 μm of spot size with the yttrium-aluminum-garnet laser at 2–5 mJ for 0.05 s.

Argon laser PI was performed with a spot size of 500 μm, for 0.4~0.5 s, and titrated power of 150~300 mW relying upon the iris response [22]. Laser beams were targeted to periphery of the iris root. The purpose was to acquire a visible contracture of the iris tissue. Approximately 24 burns were applied throughout 360° with a 2-burn space apart. To avoid the large radial vessels, care was taken throughout the laser application. PI was conducted before LI and both lasers were performed on the same one day. 

### 2.3. Dual Scheimpflug Analyzer Imaging

Dual Scheimpflug analyzer imaging was performed ahead of any contact process or the beginning of any hypotensive medications. All of the subjects underwent double Scheimpflug analyzer imaging pre-laser and one-week post-laser under consistent dim light (15 lux) in a sitting position. 

The Galilei G4 was used for all analyses. This non-contact diagnostic device is based on processed images from an incorporated rotating dual-Scheimpflug and a 20-ring Placido disk, which enables measurements of up to 100,000 points. This dual Scheimpflug analyzer has two cameras at 180 degrees apart, intended to minimize and compensate for errors regarding oblique scans. It also provides the exact pachymetry of both the central and peripheral cornea, even in situations such as trivial movements of the subjects [27]. It incorporates eye-motion compensation on the basis of characteristics of the iris and analyzes the changes in the cornea.

Corneal pachymetry of corneal zones with different diameters, specifically central (0.0 to 4.0 mm), middle (4.0 to 7.0 mm), and peripheral (7.0 to 10.0 mm), were acquired. High-order corneal aberration, including coma and trefoil, and RMS total corneal wavefront aberrations were obtained. 

Before each measurement, the central Placido rings were focused, and then the device was aligned. Then, the subject was requested to blink and look at the target of fixation. The subject was requested to watch the stimulus for 2 s to accomplish a homogeneous tear film. This process allowed the acquisition of fine-quality images and avoided corneal aberration changes following blinking [29]. Then, the person was asked not to blink during image acquisition.

### 2.4. Statistical Analysis

Corneal parameters including corneal aberration, IOP, and ACA parameters were compared before and after the laser procedures using the Linear mixed-effects model, after adjusting for age and gender. Statistical significance was considered for a *p* value of less than 0.05. All statistical analyses were conducted using SPSS software version 20.0 (SPSS, Inc., Chicago, IL, USA).

## 3. Results

Three patients were excluded before the final analysis due to poor images resulting from poor cooperation. A total of 58 eyes of 58 patients who underwent LI combined with PI were included in the final analysis. Of these, 15 subjects were men and 43 were female (Koreans). The mean age was 62.43 ± 7.38 years. There were 27 PACS eyes, 7 PAC eyes, and 24 PACG eyes. The mean spherical equivalent (SE) was 0.78 ± 1.55 D, and the baseline central corneal thickness (CCT) was 553.48 ± 37 μm. The baseline white-to-white (WTW) from the nasal to the temporal limbus was 11.62 ± 0.32 mm, and the WTW from the superior to the inferior limbus was 11.59 ± 0.32 mm (Table 1).

The central anterior chamber depth (ACD) increased significantly after LI combined with PI from a baseline of 2.09 ± 0.25 mm to 2.12 ± 0.23 mm at one week (*p* = 0.001) and to 2.10 ± 0.23 mm at one year (*p* = 0.018). The anterior chamber volume (ACV) also increased significantly after the laser procedure from a baseline of 80.72 ± 12.34 mm^3^ to 85.36 ± 14.57 mm^3^ at one week (*p* < 0.0001) and to 83.00 ± 12.17 mm^3^ at one year (*p* = 0.013). The baseline IOP was 15.97 ± 4.20 mmHg, and the IOP decreased significantly after LI plus PI to 13.77 ± 2.30 mmHg at one week (*p* < 0.0001) and to 13.73 ± 2.63 mmHg at one year (*p* < 0.0001). ACA from the superior and nasal region increased significantly after the laser procedure at 1 week and at 1 year (all *p* < 0.011). ACA from the temporal region showed a borderline significant increase at 1 week (*p* = 0.058), but it showed a significant increase at 1 year after the laser procedure (*p* = 0.038). ACA from the inferior region did not show a significant increase at both 1 week and at 1 year after the laser procedure, although the values seem larger than the baseline (all *p* > 0.05, Linear mixed-effects model) (Table 2).

Corneal aberration was assessed by RMS total wavefront aberrations. The baseline total corneal aberration was 1.09 ± 0.45 um, and it was 1.18 ± 0.66 um at one week and 1.05 ± 0.33 um at one year after LI combined with PI. There were no significant changes after the laser procedure in total corneal aberration until 1 year (*p* = 0.285 at one week, *p* = 0.562 at one year). The baseline total Coma was −0.02 ± 0.43 um, and it was −0.04 ± 0.50 um at one week and −0.01 ± 0.42 um at one year after LI combined with PI. There were no significant changes after the laser procedure in total Coma until one year (*p* = 0.553 at one week, *p* = 0.627 at one year). Each vertical coma and horizontal coma also showed no significant change after LI plus PI at both one week and one year (all *p* > 0.05). The baseline total Trefoil was −0.12 ± 0.45 um, and it was −0.09 ± 0.39 um at one week and −0.10 ± 0.32 um at one year after LI combined with PI. There were no significant changes after the laser procedure in total Trefoil until one year (*p* = 0.598 at one week, *p* = 0.761 at one year). Each vertical trefoil and oblique trefoil also showed no significant change after LI plus PI at both one week and one year (all *p* > 0.05). The baseline SE was 0.78 ± 1.55 D, and it was 0.76 ± 1.84 D at 1 one week and 0.75 ± 1.88 D at one year after LI plus PI. There were no significant changes in SE after the laser procedure until one year (*p* = 0.601 at one week, *p* = 0.87 at one year, Linear mixed-effects model) (Table 3).

CCT showed no significant changes at either one week or one year after LI combined with PI (all *p* > 0.05). Pachymetry from all central, middle, and peripheral zones also did not demonstrate significant changes at either one week or one year after the laser procedure (all *p* > 0.05). There were also no significant changes in the corneal volume at one week or one year after LI plus PI (all *p* > 0.05, Wilcoxon signed-rank test) (Table 4).

There were no serious complications observed after LI combined with PI in the current study. There were a few cases of hemorrhage at the iris during LI, but none were observed during PI. Transient AC inflammation was noticed in some subjects, but it was well managed by medical treatment.

**Figure 1:**
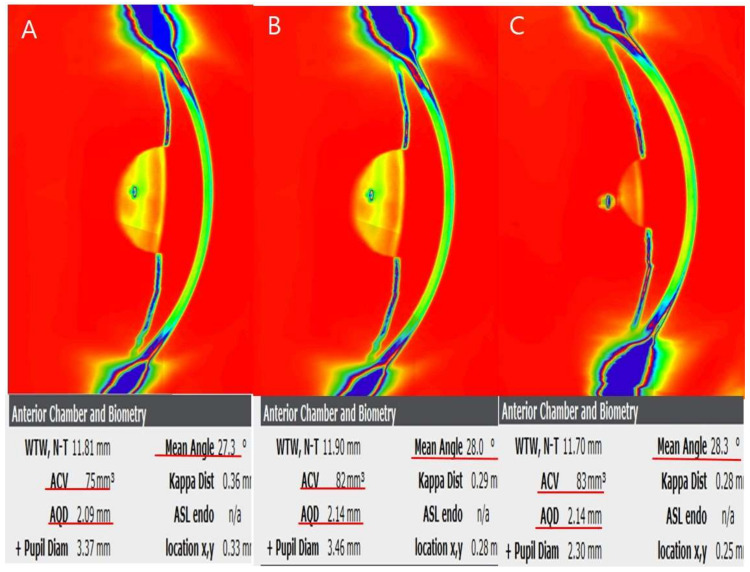
A representative case of a primary angle-closure glaucoma (PACG) patient who underwent laser iridotomy (LI) combined with peripheral iridoplasty (PI) before and after the laser procedure. The “Eye Metrics” map with an inversed, colorized image for visualizing the peripheral angle is shown. The map demonstrates anterior chamber angle parameters, anterior and posterior axial curvatures, and pachymetry ((**A**): pre-laser, (**B**): post-laser at 1 week, (**C**): post-laser at 1 year). The central anterior chamber depth (AQD, distance between corneal endothelium and anterior surface of the lens) increased from 2.09 mm (**A**) to 2.14 mm (**B**), and the anterior chamber volume increased from 75 mm^3^ (**A**) to 82 mm^3^ (**B**) after LI plus PI at 1 week in this patient. The mean anterior chamber angle also increased from 27.3° (**A**) to 28.0° (**B**). At one year after LI combined with PI, all of the ACA parameters stayed widened compared to baseline, including the ACD at 2.14 mm, the ACV at 83 mm^3^, and the mean ACA at 28.3° (**C**).

A representative case of a PACG subject before and after LI combined with PI is shown in Figure 1. The “Eye Metrics” map with an inversed, colorized image for visualizing the peripheral angle is shown in Figure 1. The map demonstrates the AC angle parameters including the ACD, ACV, and mean ACA. This 62-year-old female had a baseline central ACD (AQD, distance between corneal endothelium and anterior surface of the lens) of 2.09 mm and an ACV of 75 mm^3^, and a mean ACA of 27.3° (Figure 1A). At one week after LI plus PI, the ACD increased to 2.14 mm, the ACV to 82 mm^3^, and the mean ACA to 28.0° (Figure 1B). At one year after LI combined with PI, all of the ACA parameters stayed widened compared to the baseline, including the ACD at 2.14 mm, the ACV at 83 mm^3^, and the mean ACA at 28.3° (Figure 1C).

## 4. Discussion

In the present study, we demonstrated that angle parameters including the ACD, ACA, and ACV were significantly increased after LI combined with PI until a long-term follow-up of one year in PAC spectrum eyes. It shows the effectiveness of LI combined with PI on angle parameters in PAC spectrum eyes even for a long period of one year. The corneal parameters including high-order aberrations and pachymetry from the central, middle, and peripheral zones showed no significant changes after LI plus PI until one year of follow-up. Corneal topographic parameters including corneal aberration after LI combined with PI have not been reported after a long period, other than corneal endothelial density. We used a dual Scheimpflug analyzer to evaluate both corneal and angle parameters, which have not been reported before, particularly after a long period. 

Previous studies evaluating long-term observations of angle parameters involved only LI and not LI combined with PI. The Zhongshan Angle-Closure Prevention Trial conducted in China observed patients up to 18 months after LI [30]. They found that the angle width of LI-treated eyes increased significantly at two weeks, remained stable for six months, but decreased significantly at 18 months in PAC suspects [30]. Another study performed in South Korea on PAC suspects [31] found that the ACA tended to be narrowed at 18 months after LI despite relief of the pupillary block. Another study with a long-term follow-up of 41–54 months in South Korea revealed that ACA widened in the pupillary block and thick peripheral iris groups, but not in the plateau iris configuration and exaggerated lens vault groups after LI alone in PAC spectrum eyes [32]. These results also support our previous study results [14,18] that LI was able to relieve only the pupillary block and combined mechanisms other than just a pupillary block may contribute to PAC, especially in Asians, where PACG is more prevalent [1,2,3,4]. The changes in the ACA parameters may seem small in magnitude, but considering that these changes take place in a very small structure of the eye, other studies also show small changes. For example, the previous study by Lee et al. [31] performed in South Korea showed the baseline ACD of 2.11 ± 0.18 mm, and after only LI, ACD was 2.09 ± 0.18 mm at 18 months post-laser, which are similar values with the present study, only that our study showed a significant increase even at 1 year after LI combined with PI (2.10 ± 0.23 mm, and baseline of 2.09 ± 0.25 mm). PI is considered to be capable of relieving other mechanisms of PAC except for pupillary block. Therefore, LI combined with PI may be more effective than LI alone, particularly in Asians, as shown by our previous studies [14,18] and the present study. All ACA parameters maintained significant opening compared to baseline during the one year of follow-up in this study. These long-term follow-up assessments of LI combined with PI were initially investigated in the present study. Previous studies [30,31,32] used anterior-segment optical coherence tomography for angle assessment, whereas we used a dual Scheimpflug analyzer and could assess the circumferential mean angle and the ACA in four quadrants, not just horizontal angles, which was another unique feature of the present study. 

Previous long-term studies mainly inspected corneal endothelial density as a corneal parameter after LI or PI. A study by Kumar [33] showed that corneal endothelial density decreased over three years in PACS eyes after LI with sequential argon-Nd:YAG. However, there was no significant difference compared to untreated fellow eyes. In another study that evaluated corneal endothelial density over seven longitudinal years after LI with argon-Nd:YAG, a decrease in the corneal endothelial density was observed, but it was small during the first year and negligible after one year in PAC or PACS [34]. Another study that used only a YAG laser for LI in PACS, reported that corneal endothelial density decreased over time, principally due to aging, and LI did not seem to induce clinically significant corneal endothelial damage during the 72 months after treatment [35]. We used an argon laser for PI, and sequential argon-Nd:YAG LI was performed on the same day. A certain laser energy power is required for the peripheral iris during LI or PI. Since the peripheral endothelium of the cornea is inside or very adjacent to the “line of fire”, a theoretical risk concerning endothelial cell loss exists [36]. Müller et al. inspected corneal endothelial density in PI eyes compared to the fellow untreated eyes for an average of 30 months (1~96 months). They found a certain range of differences between the patients. Most of them did not show significant differences in central endothelial cell density between the treated and control eye [36]. They concluded that PI seems to be a safe treatment regarding corneal endothelial effects.

The periphery of the cornea is where the laser is applied during LI and PI, which is also the thickest portion of the entire cornea [37,38]. We used minimal laser energy during PI, just enough to induce visible contraction of the iris tissue. Moreover, the laser energy is markedly less than that for LI. However, the effect of LI combined with PI on peripheral corneal thickness has not been studied before. Using a dual Scheimpflug analyzer, pachymetry could be evaluated in three zones, including the central, middle, and periphery. In the present study, pachymetry values from all three zones showed no significant changes for one year after LI plus PI, notably in the peripheral zone. CCT and corneal volume also did not show significant changes over one year of follow-up in the present study. Since there were no previous studies regarding corneal thickness after LI combined with PI except for our previous study with short-term results of one week [39], which also showed no significant changes after the laser procedure, our results may be a good reference for clinicians.

Corneal aberrations influence the optical quality of vision. In addition to low-order aberrations including hyperopia, myopia, and regular astigmatism, high-order aberrations also comprise approximately 10% of the total corneal aberrations [26]. Among several high-order aberrations, coma and trefoil are of clinical importance and interest. High-order aberrations are associated with halo or glare in daily life. Highly viscous (0.3% concentration) sodium hyaluronate eye drops have been reported to increase high-order aberrations in normal eyes and in contact lens wearers [40,41]. Corneal high-order aberrations could change in an individual after using dense artificial tears, even without any intervention such as laser procedures. However, in the present study, the total corneal aberration or trefoil and coma showed no significant changes after LI combined with PI during the follow-up period of one year. The findings suggest that LI plus PI may not impair the optical quality of vision regarding corneal aberration.

The possible complications of PI may be transient IOP elevation, prolonged uveitis, iris hemorrhage, and a transient atonic pupil, which are well-controlled with conservative therapy and without long-term consequences [42]. In the present study, there were no serious complications observed during the follow-up period of one year after LI combined with PI. Hemorrhage at the iris was noticed in a few subjects during LI but none during PI. During the PI procedure, the laser was applied carefully at minimum power to accomplish visible contraction of the iris. The coagulation of the iris was prevented, as excessive laser application may induce the coagulative necrosis of the iris vessels. Hence, additional PI with LI may not have resulted in extra complications in comparison with LI alone in the current study.

There were several limitations to our study. One of them was associated with the hospital-based design, rather than a population-based study, and was carried out at a referral hospital of the province. This study was prospective, but the included subjects may not represent all PAC spectrum eyes. The relatively small number of included eyes in the present study should be addressed. However, 58 eyes of 58 subjects may still be sufficient to observe tendencies during long-term follow-up after a single procedure. The current study observed patients for one year, but longer follow-ups may be needed to evaluate other corneal changes. The persistency of the widening of the ACA after LI combined with PI needs to be seen for a longer period. Lastly, this study included only Asian (Korean) patients. Considering the anatomical structural difference and the prevalence of PACG in Asians [1,2,3,4], the results of our study may not apply to other ethnic populations. Nonetheless, the present study is unique in that all participants were East Asians (Korean), and all subjects underwent the same LI combined with PI. Therefore, this study provides long-term results on the laser procedure and the underlying mechanism of PAC. Finally, the absence of a masked evaluation by at least two experimenters could be a limitation. However, there is an advantage in that diagnosis and assessment throughout the study could be performed with consistent definition criteria by a single glaucoma specialist.

## 5. Conclusions

In conclusion, LI combined with PI significantly improved ACA parameters and persisted over a long-term follow-up of one year. Corneal parameters such as high-order aberrations and pachymetry in the central, middle, and peripheral zones were not influenced by LI plus PI until 1 year, as assessed by a dual Scheimpflug analyzer. The present study has a significant meaning in that it provides objective data concerning corneal topographic parameters along with angle parameters after LI in combination with PI in long-term observations of all PAC spectrum eyes, which has not been reported before. LI combined with PI may be an effective method for PAC spectrum eyes without corneal topographical changes. Further population-based studies with large numbers and longer follow-up are needed to reach definitive conclusions.

## Figures and Tables

**Table 1:** Baseline characteristics of the included subjects with shallow anterior chamber treated with peripheral laser iridotomy combined with iridoplasty.

Baseline Characteristics	Values
Number of subjects	58 eyes (58 patients)
Mean age	62.43 ± 7.38 years
Gender (Male: Female)	15 eyes: 43 eyes
Diagnosis	
PACS	27 eyes
PAC	7 eyes
PACG	24 eyes
Ocular factors	
SE	0.78 ± 1.55 D
CCT	553.48 ± 37 μm
WTW N-T	11.62 ± 0.32 mm
WTW S-I	11.59 ± 0.32 mm

Values represent mean ± mean deviation. Abbreviations: PACS primary angle closure suspect, PACG primary angle closure glaucoma, AACG acute angle closure glaucoma, SE spherical equivalent, CCT central corneal thickness, WTW N-T white to white from nasal to temporal limbus, WTW S-I white to white from superior to inferior limbus.

**Table 2:** Changes in the anterior chamber angle parameters post laser iridotomy combined with iridoplasty.

Parameters	Before Laser	After 1 Week	*p* Values	After 1 Year	*p* Values
IOP (mmHg)	15.97 ± 4.20	13.77 ± 2.30	**<0.0001** ^†^	13.73 ± 2.63	**<0.0001** *
Central ACD (mm)	2.09 ± 0.25	2.12 ± 0.23	**0.001** ^†^	2.10 ± 0.23	**0.018** *
ACV (mm^3^)	80.72 ± 12.34	85.36 ± 14.57	**<0.0001** ^†^	83.00 ± 12.17	**0.013** *
AC angle S (degree)	24.70 ± 3.64	26.48 ± 4.96	**0.004** ^†^	26.60 ± 5.61	**0.003** *
AC angle I (degree)	29.57 ± 6.84	30.67 ± 5.53	0.11 ^†^	29.84 ± 4.84	0.579 *
AC angle N (degree)	25.64 ± 3.13	26.42 ± 3.13	**0.011** ^†^	26.59 ± 3.88	**0.011** *
AC angle T (degree)	26.29 ± 3.89	26.98 ± 3.70	0.058 ^†^	27.07 ± 4.17	**0.038** *

Values represent mean ± mean deviation. Abbreviations: IOP intraocular pressure, ACD anterior chamber depth, ACV anterior chamber volume, AC anterior chamber, S superior, I inferior, N nasal, T temporal; ^†^ Baseline vs. 1 week or * baseline vs. 1 year: linear mixed-effects model, adjusting for age and gender. Bold font indicates significant *p*-values (*p* < 0.05).

**Table 3:** Changes in total corneal aberration and total corneal power post laser iridotomy combined with iridoplasty.

Corneal Aberrations	Before Laser	After 1 Week	*p* Values	After 1 Year	*p* Values
Total corneal aberration (μm)	1.09 ± 0.45	1.18 ± 0.66	0.285 ^†^	1.05 ± 0.33	0.562 *
Vertical Coma (μm)	−0.06 ± 0.28	−0.06 ± 0.35	0.847 ^†^	−0.04 ± 0.27	0.522 *
Horizontal Coma (μm)	0.04 ± 0.36	0.02 ± 0.38	0.431 ^†^	0.03 ± 0.33	0.827 *
Total Coma (μm)	−0.02 ± 0.43	−0.04 ± 0.50	0.553 ^†^	−0.01 ± 0.42	0.627 *
Vertical Trefoil (μm)	−0.03 ± 0.32	−0.01 ± 0.34	0.691 ^†^	−0.03 ± 0.22	0.894 *
Oblique Trefoil (μm)	−0.08 ± 0.23	−0.07 ± 0.24	0.729 ^†^	−0.07 ± 0.22	0.788 *
Total Trefoil (μm)	−0.12 ± 0.45	−0.09 ± 0.39	0.598 ^†^	−0.10 ± 0.32	0.761 *
SE (D)	0.78 ± 1.55	0.76 ± 1.84	0.601 ^†^	0.75 ± 1.88	0.87 *

Values represent mean ± mean deviation. Abbreviations: Ant. Anterior, Post. Posterior, TCP total corneal power, D Diopter, Mid middle, Pph periphery, SE spherical equivalent; ^†^ Baseline vs. 1 week or * baseline vs. 1 year: linear mixed-effects model, adjusting for age and gender.

**Table 4:** Changes in corneal thickness post laser iridotomy combined with iridoplasty.

Parameters	Before Laser	After 1 Week	*p* Values	After 1 Year	*p* Values
CCT (μm)	553.48 ± 37.00	555.42 ± 38.30	0.76 ^†^	552.53 ± 36.45	0.293 *
Pachymetry Central (μm)	564.00 ± 37.21	566.01 ± 37.93	0.727 ^†^	563.36 ± 36.63	0.346 *
Pachymetry Mid (μm)	614.83 ± 36.58	616.98 ± 39.24	0.679 ^†^	614.66 ± 36.69	0.49 *
Pachymetry Pph (μm)	696.89 ± 49.30	701.36 ± 50.96	0.543 ^†^	689.54 ± 43.35	0.167 *
Corneal volume (mm^3^)	32.17 ± 1.92	32.30 ± 2.06	0.606 ^†^	32.12 ± 1.92	0.342 *

Values represent mean ± mean deviation. Abbreviations: CCT central corneal thickness, Mid middle, Pph periphery; ^†^ Baseline vs. 1 week or * baseline vs. 1 year: linear mixed-effects model, adjusting for age and gender.

## Data Availability

Not applicable.
